# One-stage versus two-stage revision of the infected knee arthroplasty - a randomized multicenter clinical trial study protocol

**DOI:** 10.1186/s12891-021-04044-8

**Published:** 2021-02-12

**Authors:** Martin Lindberg-Larsen, Anders Odgaard, Charlotte Fredborg, Henrik Morville Schrøder, on behalf of the One-stage vs Two-stage Collaboration Group, Jens Bagger, Thomas Bruno Lind-Hansen, Anders Kunov, Per Hansen, Jeppe Lange, Andreas Kappel, Kim Hansen, Claus Emmeluth

**Affiliations:** 1 Department of Clinical Research, University of Southern Denmark, Odense, Denmark; 2 Orthopaedic Research Unit, Department of Orthopaedic Surgery and Traumatology, Odense University Hospital, Odense, Denmark; 3 Department of Orthopaedic Surgery and Traumatology, Rigshospitalet - Copenhagen University Hospital, Copenhagen, Denmark; 4 Intensive Care Unit, Copenhagen University Hospital Rigshospitalet-Glostrup, Copenhagen, Denmark; 5 Department of Orthopaedic Surgery, Næstved Hospital, Næstved, Denmark

**Keywords:** Knee arthroplasty, Infection, Revision, One-stage, Two-stage, PROM

## Abstract

**Background:**

A two-stage prosthesis exchange procedure has been the gold standard in surgical treatment of the chronically infected knee arthroplasty so far. This includes 2 surgeries/hospitalizations and an interim period of 2–3 months between surgeries with impaired health, functional status and quality of life of the patients. A one-stage exchange procedure holds many obvious advantages compared to the two-stage approach, but outcomes of a one-stage versus two-stage procedures have never been investigated in a randomized clinical trial. The purpose of this study is primarily to investigate time-adjusted differences in functional status of patients after one-stage versus two-stage revision. Secondary, to report time-adjusted differences in quality of life, complications (including re-revisions due to infection) and mortality.

**Methods:**

This study is a pragmatic, multi-center, randomized, non-inferiority trial comparing one-stage versus two-stage revision of the infected knee arthroplasty. Seven Danish hospitals are currently participating in the study, but additional hospitals can enter the study if adhering to protocol. Ninety-six patients will be included prospectively. Follow-up will be with PROM-questionnaires and clinical controls up to 10 years. The patients who are not able to participate in the randomized trial are followed in a parallel cohort study.

**PROM’s:**

Oxford Knee Score and EQ5D + EQ5D VAS questionnaires are completed preoperatively and sent out to the study participants at 6 weeks, 3, 6, 9, 12, 18 and 24 months as well as 5 and 10 years postoperatively. In addition a tailor made cost questionnaire on the non-treating hospital resource use, community health and social service use, travel costs, time off work and informal care are sent out.

**Discussion:**

If one of the two treatment alternatives is found superior in both domains of quality of life (both knee-specific and generic) and health economics, that treatment should be promoted. Other outcomes will open informed discussions about treatment strategies for periprosthetic knee infections.

**Trial registration:**

The randomized trial is registered on ClinicalTrials.gov with ID NCT03435679, initial release date January 31, 2018 and the cohort study is registered with ID NCT04427943, submitted January 8, 2020 and posted June 11, 2020.

## Background

The two-stage exchange procedure has been the gold standard in surgical treatment of the chronically infected knee arthroplasty so far. This includes 2 hospitalizations and 2 surgeries as well as an interim period in between the surgeries. The study group has previously investigated outcome of the surgical treatment of infected knee arthroplasties (including 215 two-stage procedures) on a nationwide basis in Denmark. The interim period between surgeries was 3 months on average with an infection eradication rate of 70% [1]. Furthermore, these challenging procedures were performed in 25 different orthopaedic centres in Denmark and with variable diagnostic approaches [1, 2]. A recent review from 2019 reported data from 18 studies (*n* = 10–177) on two-stage procedures performed from 2000 to 2018 and found that the average infection eradication rate was 85% (range 54–100%) [3]. Almost all studies included in the review were single-center studies (*n* = 10–177), but the results confirm that a two-stage approach is associated with risk of re-infection and is not a perfect treatment option [3].

The patients are more or less immobilized during the interim period between the 2 stages in the two-stage approach. This may be associated with a potentially increased risk of associated morbidity and reduced quality of life for the patient. Furthermore, the economic burden for the hospitals is high, when treating periprothetic joint infections in two-stages [4]. The 2 hospitalizations and 2 procedures are costly for the hospital, but the longer sick leave, trouble getting around during interim period and the greater need of help may also be costly for the patients.

There are potential benefits for patients treated with a one-stage approach as they only have to go through surgery and rehabilitation once with shorter total length of hospital stay. Promising results after one-stage prosthesis exchange procedures have been reported from retrospective single-center studies with highly selected patients and strict surgical protocols [5–7]. A review of 14 studies (*n* = 14–130) performed from 1992 to 2017 found an average infection eradication rates of 87% (range 67–100%) after one-stage procedures [3]. Data comparing functional outcomes between one- and two-stage procedures are sparse, but the existing data suggest that results are comparable [3].

We find it reasonable to hypothesize that the time-adjusted changes in functional outcome and quality of life of patients are non-inferior after one-stage surgery compared to two-stage surgery. The two treatment strategies have never been compared in a randomized clinical trial.

The aim of this study is primarily to investigate time-adjusted differences in functional status of patients within the first postoperative year after one-stage versus two-stage revision surgery in a randomized study design. Secondary, to report time-adjusted differences in functional status, quality of life, complications (including re-revisions due to infection) and mortality within 2 years, 5 years and 10 postoperatively.

## Methods

This study is a pragmatic, multi-center, two-arm, parallel group, open, randomized, non-inferiority trial with 1:1 allocation. This trial will comply with the CONSORT 2010 Statement [8] and the SPIRIT guidelines [9].

### Participants and study setting

Ninety-six participants will be included consecutively in the study. So far, seven Danish hospitals are participating in the study, but additional hospitals can enter the study if adhering to protocol. Currently participating hospitals are Odense University Hospital, Aalborg University Hospital, Copenhagen University Hospital Bispebjerg, Copenhagen University Hospital Gentofte, Naestved Hospital, Regional Hospital Horsens, Lillebaelt Hospital - Vejle, University Hospital of Southern Denmark and Rigshospitalet - Copenhagen University Hospital. Experienced revision knee surgeons perform the surgical procedures.

### Inclusion/exclusion criteria

The inclusion criteria in this study are pragmatic and wide, and the exclusion criteria are limited compared to previous studies reporting data on one-stage procedures. We chose this deliberately in order to include as many patients as possible who would have been offered a two-stage procedure as a standard treatment if not participating in the study. In this way, we hope that the results of our study will have larger clinical impact on everyday practice. As the two-stage approach is the standard procedure so far and the one-stage procedure is the “new” procedure, the participating investigators agreed that patients could only be offered a one-stage procedure if they participated in the trial and were randomized to one-stage. Inclusion/exclusion criteria are listed in Table 1.

**Table 1:** Inclusion and exclusion criteria

Inclusion criteria	Exclusion criteria
- Clinical signs of periprosthetic knee infection and indication for revision surgery - > 6 weeks from previous knee arthoplasty procedure (primary or total revision procedure) - Speak and understand Danish and able to give informed consent	- soft tissue problems requiring plastic surgery - major bone loss requiring mega/tumor-prosthesis - acute surgery due to sepsis - malignant disease with less than 2 years life expectancy - re-infection after previous two-stage procedure - bilateral knee infection

### Flow

**Fig. 1:**
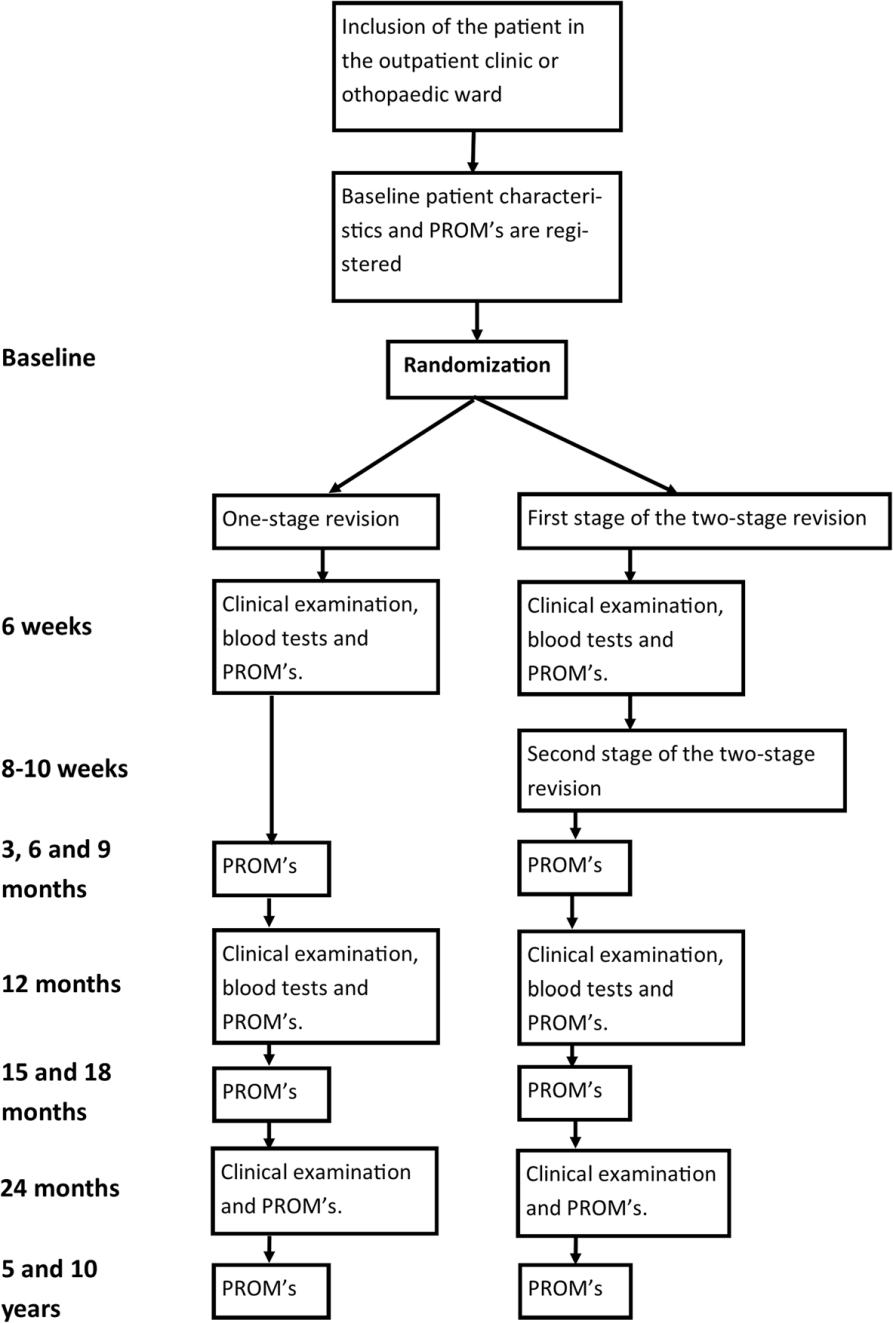
Flow-chart on inclusion, randomization and follow-up

Patients with a clinically infected knee arthroplasty requiring revision surgery, who are referred to or admitted in one of the participating hospitals, will be screened for eligibility by a knee-surgeon and subsequently offered inclusion in the study. Eligible patients will receive oral and written information regarding the study, and offered time to consider participation. If a patient is willing to participate, the site investigator ensures that the patient has read and understood all the received information, and the patient will be given the opportunity to ask questions. After written consent, the patient completes baseline questionnaires and a physical examination is performed (Fig. 1).

### Intervention

#### The one-stage procedure

- ○ 5 tissue biopsies (Kamme-Lindberg), debridement including total synovectomy, removal of prosthesis, bone cuts (preparing to accommodate the chosen prosthesis), pulse-lavage with a minimum of 3 L saline followed by 1 L saline with antibiotics (2 g Vancomycin and 240 mg Gentamycin).
- ○ A temporary prosthesis trial may be inserted to ensure stability while re-draping (not previously used, e.g. other size).
- ○ A soaked cotton gauze is placed within the wound and temporary capsule and wound closure with sutures is performed and wound is covered with temporary wound dressing.
- ○ Area around the patient is cleared by removing surgical draping, suction, pulsatile-lavage and used instruments.
- ○ **Time-out**
- ○ The surgical team puts on new sterile surgical gowns, the skin is prepared and the knee is draped as at the beginning of a new procedure.
- ○ Pulsatile-lavage with another 1 L of saline.
- ○ Implantation of new prosthesis using antibiotic-laden acrylic cement (gentamycin and/or vancomycin/clindamycin). If/when microbiological diagnosis is known, targeted intra- and postoperative antibiotics is given according to recommendations from local microbiological department.
- ○ Antibiotic regime:
  - ▪ If microbiological diagnosis is known targeted intraoperative intravenous (IV) antibiotics is given after tissue biopsies is performed.
  - ▪ Postoperative antibiotics is given for 6 weeks, with initially 2 weeks of IV treatment. If oral administration is possible, this is used for the remaining 4 weeks, otherwise the intravenous administration is continued according to local microbiological recommendations.
  - ▪ If microbiological diagnosis is unknown (first dose is given intraoperatively after tissue biopsies):
    - IV Dicloxacillin or Flucloxacillin 1 g × 4 (if allergy cefuroxim 1.5 g × 3)
    - IV Vancomycin 1 g × 2

#### The two-stage procedure

- Stage 1:
  - ○ 5 tissue biopsies, debridement including total synovectomy, removal of prosthesis removal of all cement from previous prosthesis, pulse-lavage with a minimum of 3 L water followed by 1 L water including 2 g Vancomycin and 240 mg Gentamycin.
  - ○ Antibiotic loaded cemented spacer (fixed or articulated as per surgeon preference) is implanted using antibiotic-laden acrylic cement (gentamycin and/or vancomycin/clindamycin).
  - ○ Weight bearing and brace according to surgeons choice.
  - ○ Antibiotics regime:
    - ▪ as in one-stage, but after 6 weeks of antibiotics treatment, treatment is paused for 2 weeks before stage 2 to ensure infection control (monitored clinically and paraclinically).
- Stage 2 (8–10 weeks after stage 1):
  - ○ 5 tissue biopsies, removal of spacer, debridement including total synovectomy, bone cuts (preparing to accommodate the chosen prosthesis), pulse-lavage with a minimum of 3 L water followed by 1 L water including 2 g Vancomycin and 240 mg Gentamycin.
  - ○ Implantation of new prosthesis using antibiotic-laden acrylic cement (gentamycin and/or vancomycin).
  - ○ Antibiotics regime:
    - ▪ first dose is given intraoperatively after tissue biopsies:
      - IV Dicloxacillin or Flucloxacillin 1 g × 4 (if allergy cefuroxim 1.5 g × 3)
      - IV Vancomycin 1 g × 2
    - ▪ Antibiotic treatment until results from tissue biopsies is known and if negative, treatment is suspended. If positive, treatment is adjusted according to guidance from local microbiological department.

### Patient-reported outcomes

Oxford Knee Score (OKS) and EQ5D + EQ5D vas questionnaires are completed preoperatively and sent out to the study participants at 6 weeks, 3, 6, 9, 12, 18 and 24 months as well as 5 and 10 years postoperatively.

Furthermore, a tailor-made cost questionnaire on non-treating hospital resource use such as community health and social service use, travel costs, time off work and informal care are sent to the study participants at the same times.

### Clinical follow-up

The patients are invited to physical examination and blood tests (C-reactive protein test (CRP) and white blood cell count (WBC)) in the outpatient clinic 6 weeks, 3 months, 1 year and 2 years after the one-stage procedure or the first stage of the two-stage procedure (Fig. 1).

### Primary outcome

**Area under curve (AUC; as time adjustment) for Oxford Knee Score (OKS)** within the first postoperative year is the primary outcome measure [10]. The time-course for the primary endpoint is set to 1 years postoperatively as the majority of re-infections occur within the first postoperative year (median 95 days after two-stage procedures in Denmark) [1]. OKS is a patient reported outcome measure (PROM) with a scale range from 0 (severe symptoms) – 48 (satisfactory joint function) [11]. OKS consists of 12 simple questions easily answered by the patient. There are a low number of incorrectly completed forms [11, 12], and the form can be answered in a short time. OKS has been shown to be sensitive and OKS has been translated to and validated in multiple languages [13–15].

### Secondary outcomes

#### EQ5D + EQ5D VAS

Standardized instrument for measuring generic health status, without and with a visual analogue scale, respectively [16].

#### Implant survival (re-revision rate)

Whether the patient has undergone re-revision surgery of the arthroplasty or other additional surgery to the knee due to infection or other causes within 2, 5 and 10 years postoperatively. The information will be retrieved from the Danish National Patient Register [17] and from the Danish Knee Register [18].

#### Mortality

Postoperative mortality within 90 days (after first stage of the two-stage procedure).

#### Readmission rate

Postoperative unplanned readmissions within 90 days (after first stage of the two-stage procedure).

#### Clinical outcome measures

Physical examinations will be performed at baseline and post-operatively at 6 weeks, 3 months, 1 year and 2 years.

### Knee range of motion

The number of degrees the examiner can move the knee joint through its full range of motion with no active effort from the patient (passive movement). Mobility is measured with a standard goniometer (30 cm).

### Explorative outcome measure

#### Health economic analysis

We will conduct an intention-to-treat cost-effectiveness analysis (CEA) with the endpoint incremental cost per quality-adjusted life year (QALY) gained during the 24-month study period. The statistical uncertainty of Incremental Cost-Effectiveness Ratio (ICER) will be presented in an acceptability curve. The primary outcome QALY will be derived from the randomized patients who will complete the EQ-5D-5L prior to the operation (baseline), 6 weeks, 3, 6, 9, 12, 18 and 24 months after surgery (first stage of two-stage procedure). At 6 weeks, 3, 6 and 12 months after surgery, the patients will answer a cost questionnaire to collect information on resource use. This tailor-made cost questionnaire addresses the non-treating hospital resource use such as community health and social service use, travel costs, time off work and informal care.

The CEA will be analyzed from a societal perspective. Costs related to patients’ expenditures and work-related consequences (indirect costs) will be analyzed separately and will not be part of the base case ICER. All costs will be reported in 2023 prices (or appropriate – depending on date of inclusion of last participant), and discounting will be applied as appropriate with the recommended 4% annual discount rate. This includes the interventions, additional hospitalizations, outpatient visits and any related surgical or non-surgical procedures.

### Sample size

The statistical power calculation was performed on the primary outcome measure in patients without treatment failure (per-protocol analysis), i.e. the time adjusted OKS change during the first 12 postoperative months, which is calculated as the 12-month area under curve (AUC) for Oxford Knee Score (OKS) divided by 12 months. For the calculation, one needs reasonable estimates of 1) the mean outcome of the two randomization groups, 2) the minimal clinically relevant difference between groups that would be acceptable without declaring one group inferior (the non-inferiority margin) and 3) a reasonable estimate of the standard deviation for the two groups.

#### Ad 1

Singer et al. [6] found OKS at baseline to be 12, and the postoperative values after 3, 6, 9 and 12 months were 24, 28, 30 and 31, respectively after one-stage revision procedures due to infection. From this, the 12-month AUC is 166.5 months, corresponding to a time-adjusted improvement of 166.5 months / 12 months = 13.9 OKS points. This is our estimate of the time-adjusted OKS for the 1-stage group. For the calculation, we assume that the 2-stage treatment would result in the same mean, time-adjusted OKS.

#### Ad 2

The minimal clinically important difference (MCID) for the time-adjusted OKS improvement has not been established, but it is reasonable to equate this to the otherwise used MCID for OKS, i.e. 5 [19].

#### Ad 3

The data from Singer et al. [6] does not directly allow a calculation of the AUC standard deviation, so data from Odgaard et al. [10] is used. They reported 2-year data on AUC for TKA, but 1-year data was also available on request and they showed a 1-year AUC for TKA patients of 95 months corresponding to a time-adjusted OKS improvement of 95 months / 12 months = 7.9.

The standard deviation of the AUC was 92.6 months (tabulated as 1.93 in the dataset on a 0–1 PROM scale), this equates to standard deviation for the time-adjusted OKS of 92.6 months / 12 months = 7.7.

We find it reasonable to assume, that the standard deviation for patients without treatment failure in the 1- and 2-stage groups in the current study will be similar to this, and consequently, we expect a standard deviation for the time-adjusted OKS of 7.7.

#### Sample size

Using a non-inferiority power calculation (http://powerandsamplesize.com/Calculators/Compare-2-Means/2-Sample-Non-Inferiority-or-Superiority) based on similar means of 13.9 OKS points, in both treatment groups, common standard deviation of 7.7, a non-inferiority margin of 5, an alpha of 0.025 [20], a power of 0.8, a 1:1 sampling ratio and a one-sided comparison of two means, we reach a sample size for each group of 38, i.e. a total study sample of 76. Acknowledging uncertainty of the estimates and potential drop-outs, we increased the total sample size by 25% reaching a total of 96 patients.

### Statistical analysis plan

Changes between pre- and postoperative status will be treated with paired statistics, either parametric or nonparametric depending on the nature of the data. The development of variables over time, e.g. OKS, will be analyzed by calculating the area under the curve (AUC) for the variable relative to the initial value [10], and comparisons of the between-group differences will be based on parametric statistics. Furthermore, differences between groups in risk of complications will be analyzed. Proportions in the two treatment groups will be compared using chi-squared –test or Fisher’s exact test.

Analyses will be performed based on both the ‘Intention-to-treat’ (ITT) principle and per-protocol (PP) principle. It is relevant to report outcomes of all participants regarding patient safety and complications in an ITT analysis, but it is also relevant to compare functional outcomes in patients without treatment failure in a PP analysis. Statistical significance will be judged with 95% confidence intervals presented. Missing data will be handled using multiple imputations. Analyses are planned early at 1 and 2 years after the last participant is included, medium at 5 years, and late at 10 years follow-up of last participant.

### Randomization and data collection

The random allocation sequence is computer-generated using REDCap with stratification based on age +/− 70 years and whether microbiological diagnosis is known preoperatively. The site-investigators at each site are performing randomization in REDCap shortly after enrolling a participant. The study is not blinded to either participants or investigators. All data are collected and stored digitally in a REDCap database. PROM’s are sent out and filled in digitally by the patients. Alternatively, the participants are contacted by mail or phone by the research nurse on request. Clinically outcomes are filled in the REDCap database by the site-investigators with range checks and alerts if data is not within the expected range. In case of participant drop out, the automatic REDCap PROM surveys are stopped, but clinical data are still registered for the safety analysis.

### Monitoring

Periprosthetic knee infection is a serious condition. Postoperative complications and risk of mortality are expected, but the risk of complications should not be increased by participating in the study. The safety of the participants is monitored by MLL and HMS. If the number of treatment failures (re-infections requiring re-revision or 90 days mortality) becomes twice as high in one of the allocation groups the study is terminated. However, at the earliest after inclusion of 40 participants and the number of failures should be minimum 10. If this happens, it will be reported to The Regional Committees on Health Research Ethics for Southern Denmark as a serious adverse event. Interim analysis is performed after inclusion of 40 participants and annually after this.

### Timeline

Recruitment of participants started February 2018. Based on a patient acceptance ratio of 20% and the current number of infected knees screened and included in the participating centers so far, inclusion is expected to last up to 9 years in total as a conservative estimate. However, additional centers are expected to enter the study with resulting reduction in the inclusion time.

## Discussion

The study group has designed this randomized clinical trial to investigate outcome of a one-stage revision approach compared to the more commonly used two-stage approach in patients with periprosthetic knee infection. If one of the two treatment alternatives is found superior in both domains of quality of life (both knee-specific and generic) and health economics, that treatment should be promoted. Other outcomes will open informed discussions about treatment strategies for periprosthetic knee infections.

The study is dimensioned based on the primary end point of this study which is the time-adjusted improvement in Oxford Knee Score within the first postoperative year. This is a functional outcome based on patient reported outcomes. We do acknowledge that patient safety is as important and we report risk of infection recurrence, re-revision, readmission and mortality as well. Furthermore, quality of life and the socio-economic aspect is important and this is also analysed in this study.

To our knowledge, this is the first study protocol of a randomized clinical trial on one-stage versus two-stage revision of periprosthetic knee infection. Most previous studies investigating outcomes of one- and two-stage revisions of periprosthetic knee infections are single-center studies and retrospective [3].

A prospective cohort study on one-stage revisions of chronically infected hip arthroplasties has been performed finding promising low risk of re-revision due to infection of 9% [21]. Furthermore, a study protocol on a multicenter randomized clinical trial investigating outcomes of one- and two-stage revisions of periprosthetic hip infections from England and Wales has been published [22]. Stange et al. - similar to our study, use a pragmatic approach with a functional outcome as primary outcome in their study protocol. Results from this study have not been published yet.

So far, it has been possible to include 20% of the patients with surgically demanding periprosthetic knee infections from the including centres in this randomized clinical trial. Hence, we have from 2020 conducted a prospective cohort study (RIKA cohort study, ClinicalTrials.gov Identifier NCT04427943) without intervention including the patients who are not able to participate in the RCT. In this study we investigate the same outcomes as in the RCT in order to obtain high quality prospective data on function, quality of life and complications after all types of surgical interventions performed due to periprosthetic knee joint infection.

Based on the data from both the RCT study and the cohort study it will be possible to evaluate the surgical treatment overall and it will be possible to evaluate whether the patients included in the randomized trial are representative. We are convinced that the data from our studies can help to standardize and streamline surgical strategies in the treatment of periprosthetic knee joint infections in order to improve outcome for patients.

### Trial status

Thirty-three participants are enrolled in the RCT study since enrolment of first participant March 6, 2018 and 24 participants are enrolled in the cohort study since enrolment of first participant February 8, 2020.

## Data Availability

The datasets used and/or analysed during the current study are available from the corresponding author on reasonable request.

## Abbreviations

TKA: Total knee arthroplasty
RCT: Randomized controlled trial
PROM: Patient reported outcome measure
OKS: Oxford Knee Score
AUC: Area under curve
CRP: C-reactive protein test
WBC: White blood cell count
CEA: Cost-effectiveness analysis
QALY: Quality-adjusted life year
ICER: Incremental Cost-Effectiveness Ratio
MCID: Minimal clinically important difference
ITT: Intention-to-treat
PP: Per-protocol
